# Mineral-mediated carbohydrate synthesis by mechanical forces in a primordial geochemical setting

**DOI:** 10.1038/s42004-020-00387-w

**Published:** 2020-10-16

**Authors:** Maren Haas, Saskia Lamour, Sarah Babette Christ, Oliver Trapp

**Affiliations:** 1 Department of Chemistry and Pharmacy, Ludwig-Maximilians-University, Butenandtstr. 5-13, 81377 Munich, Germany; 2 Max-Planck-Institute for Astronomy, Königstuhl 17, 69117 Heidelberg, Germany

**Keywords:** Origin of life, Monosaccharides, Biogeochemistry

## Abstract

The formation of carbohydrates represents an essential step to provide building blocks and a source of chemical energy in several models for the emergence of life. Formaldehyde, glycolaldehyde and a basic catalyst are the initial components forming a variety of sugar molecules in the cascade-type multi-step formose reaction. While numerous side reactions and even deterioration can be observed in aqueous media, selective prebiotic sugar formation is feasible in solid-state, mechanochemical reactions and might have occurred in early geochemistry. However, the precise role of different basic catalysts and the influence of the atmospheric conditions in the solid-state formose reaction remain unknown. Here we show, that in a primordial scenario the mechanochemical formose reaction is capable to form monosaccharides with a broad variety of mineral classes as catalysts with only minute amounts of side products such as lactic acid or methanol, independent of the atmospheric conditions. The results give insight into recent findings of formose sugars on meteorites and offer a water-free and robust pathway for monosaccharides independent of the external conditions both for the early Earth or an extra-terrestrial setting.

## Abstract (web summary)

Formose chemistry has been proposed as a prebiotic route to carbohydrates, but simple mechanisms that impart selectivity are few. Here a range of minerals thought to have existed in the Hadean environment are shown to bias the product distribution of mechanochemical carbohydrate synthesis towards pentoses and hexoses.

## Introduction

Prebiotic chemistry aims to find pathways to important organic reactants that are compatible with the harsh environmental conditions present on early Earth. Over time these basic organic molecules evolved into a diverse set of molecules essential for life, metabolism, genetic information and compartmentalisation. These biologically relevant molecules include amino acids, peptides, lipids, nucleotides and sugars. For the latter, one of the plausible reaction pathways next to Eschenmoser’s glyoxylate scenario is the formose reaction^1–3^, which was first discovered by Butlerow in 1861^4^. The formation of sugars from formaldehyde is generally catalysed by calcium hydroxide, but mineral catalysis has also been suggested for prebiotic pathways on the early Earth^5,6^. Due to the discovery of ribose and other sugars in meteorites, an extra-terrestrial formose reaction during aqueous alteration of the parent body and delivery to the early Earth has also been proposed^7^.

**Fig. 1:**
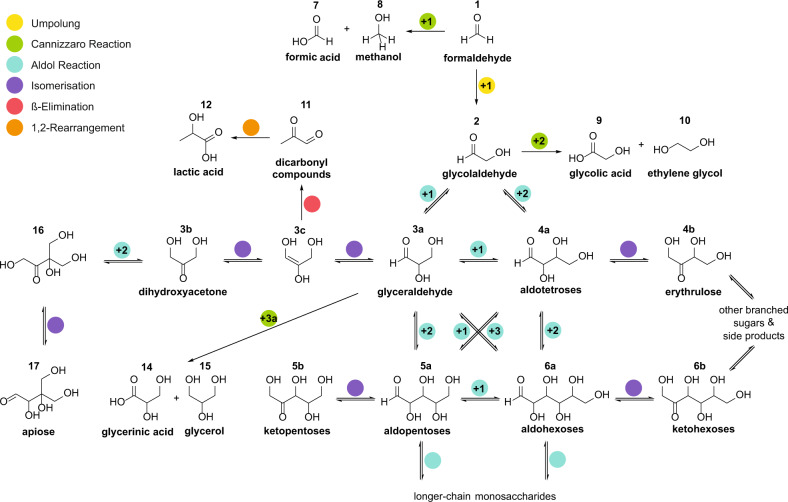
Excerpt of the formose reaction network. Overview of reaction pathways and competing reactions generating monosaccharides and related side products from formaldehyde and a basic catalyst.

In solution, the catalytic activity mostly depends on two factors: the basicity and the metal ion which can coordinate to the oxygen atoms of the sugar compounds^8,9^. Formaldehyde **1** reacts in the first step via an umpolung step to form glycolaldehyde **2** (Fig. 1). The mechanism of this dimerisation has not been completely elucidated, yet several possible pathways have been discussed^10–12^. The dimerisation can be facilitated either photochemically via radical reactions^13^ or by the use of umpolung catalysts such as thiazolium salts similar to vitamin B_1_, which is involved in the modern metabolism of sugar derivatives^14^. In the competing Cannizzaro reaction, formaldehyde is converted to formic acid **7** and methanol **8**. As soon as **2** is formed, enolisation is possible and aldol as well as retro-aldol reactions build up longer-chain monosaccharides **3–6**. Therefore, the circumvention of the umpolung step by the addition of enolisable catalysts or **2** and other sugar products provides an additional approach to increase reactivity^15^. Other side reactions are known to occur like the β-elimination yielding dicarbonyl compounds **11**, that can further undergo a 1,2-rearrangement and build up lactic acid **12**^16^.

The instability of the products under aqueous, alkaline reaction conditions and the deterioration of the products forming insoluble, undefined polymeric substances constitute the major drawbacks of this reaction^17–19^. To prevent this, we previously probed a solvent-free variation of this reaction and observed a mechanochemical acceleration and alteration of product formation^20^. Albeit the mechanochemical reaction is slower compared to the aqueous reaction, it gains from higher selectivity and less decomposition. From a prebiotic chemistry perspective, the mechanical energy required could come from geological processes like weathering, erosion and diagenesis. Synthesis in between mica sheets induced by their movement due to temperature differences or ocean currents has also been suggested^21^. Other sources for mechanical energy include tectonics, which might have even been present on the Earth as early as about 4 Ga ago^22^ and can likewise occur on asteroids^23^. In addition to possibly introducing organic matter, impact events also lead to a mechanical energy input and under these conditions the synthesis of amino acid derivatives as well as the stability of glycolaldehyde have been shown^24,25^. For laboratory purposes ball milling is the standard procedure for performing mechanochemical reactions^26^, which have been applied to mineral catalysis and even gaseous reactants^27–29^. Here we describe the broad applicability for the mechanochemical sugar synthesis and assess the prebiotic viability of the reaction under diverse mineral and atmospheric conditions. A range of minerals is shown to catalyse the monosaccharide formation from glycolaldehyde **2** and selectively influence the product distribution. Formaldehyde **1** is adsorbed on minerals which catalyse its reaction with **2** building trioses to heptoses while only marginally producing side products. An umpolung of **1** is achieved with thiazolium salts. Finally, the tolerance of the reaction towards gas phases and low temperatures is demonstrated.

## Results

### Mineral catalysis

Starting from the classical formose catalyst calcium hydroxide and its corresponding mineral portlandite, we wanted to explore the catalytic activity of different mineral classes. It is not completely certain which minerals are of significance in this context, but a summary of possible Hadean minerals has been given by Hazen^30^, which was used as a guideline in the selection of minerals. Extending the range of the previously reported three minerals (portlandite, brucite and sodium mont-morillonite clay)^20^, 18 additional minerals were used.

**Fig. 2:**
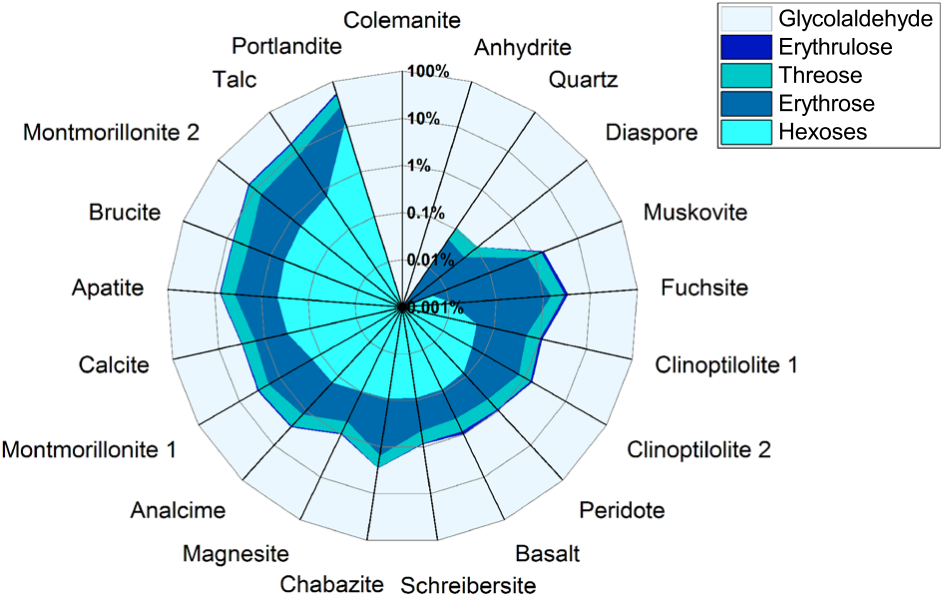
Mineral-catalysed mechanochemical monosaccharide formation. Comparison of product distribution observed after ball milling of glycolaldehyde and the different minerals catalysts (20 mol% catalyst, head space gas: air, oscillatory ball mill, 90 min, 30 Hz). Analysis of products after derivatisation as silylated *O*-ethyl oximes by GC–MS. Depicted are mean values of product ratio percentages, individual data points are omitted for clarity (for detailed tabulated data see Supplementary Table 4).

The minerals were tested with **2** as starting material in an oscillatory ball mill at 30 Hz for 90 min. Representatives of hydroxides, carbonates, sulphates, silicates, micas, zeolites, clays, olivines, phosphates, phosphides and borates were used as catalysts. Apart from anhydrite (sulphate mineral) and colemanite (borate mineral) all minerals showed catalytic activity yielding the aldol products of **2** with even-numbered carbon chain length, tetroses **4** and hexoses **6** (Fig. 2). Even the meteoritic material schreibersite did catalyse the reaction. Previously, it was shown that corroded schreibersite produced an alkaline medium in water and could therefore catalyse this reaction^31^. In the mechanochemical setting, the reaction took place even without the addition of water. Meteorites can in fact also have an extensive variety of mineral species including quartz, talc, olivines, carbonates and schreibersite from the above tested mineral selection^30^. Therefore, the solid-state formose reaction is not only limited to early Earth scenarios in the context of the origin(s) of life, but could also be possible in extra-terrestrial settings. So far the observation of ribose and other sugars in meteorites has been related to formose-type reactions or photochemistry before accretion of the parent body or during the aqueous alteration phase^7,32,33^.

The product distribution and the conversion proved to be very dependent on the mineral composition. While **4** was formed only in traces for quartz and diaspore, it made up more than 40% of the formose reaction product for portlandite. A higher conversion did not generally correspond to more **6** formation. Calcite and fuchsite were similar in conversion, but while fuchsite produced only traces of **6**, calcite yielded 0.3%. Fuchsite and muscovite are both micas, but the chromium-rich fuchsite showed nearly double the conversion. For the two montmorillonite clays, that were obtained from different sources and vary in the calcium, magnesium and sodium content, also a significant divergence in conversion was observed. In general, more tetroses than hexoses were formed and more aldoses than ketoses, but the ratios are tremendously influenced by the catalyst. The aldotetroses/erythrulose ratio varied from a value of 5 for basalt to 53 for chabazite. However, not only the equilibrium between aldoses and ketoses can be influenced by the catalyst, but also the prevalent diastereomer can be chosen. The erythrose/threose ratio ranged from 0.71 for brucite to 1.23 for montmorillonite 1, changing the observed main product within the aldotetroses.

Even though the majority of the milling experiments was performed at room temperature, sugar formation still occurred, albeit to a lesser degree, when performing the reaction under cooling with liquid nitrogen. Hence, the mechanochemical reaction even tolerates harsher environments and a range of thermal conditions as well.

### Formaldehyde incorporation and dimerisation

**Fig. 3:**
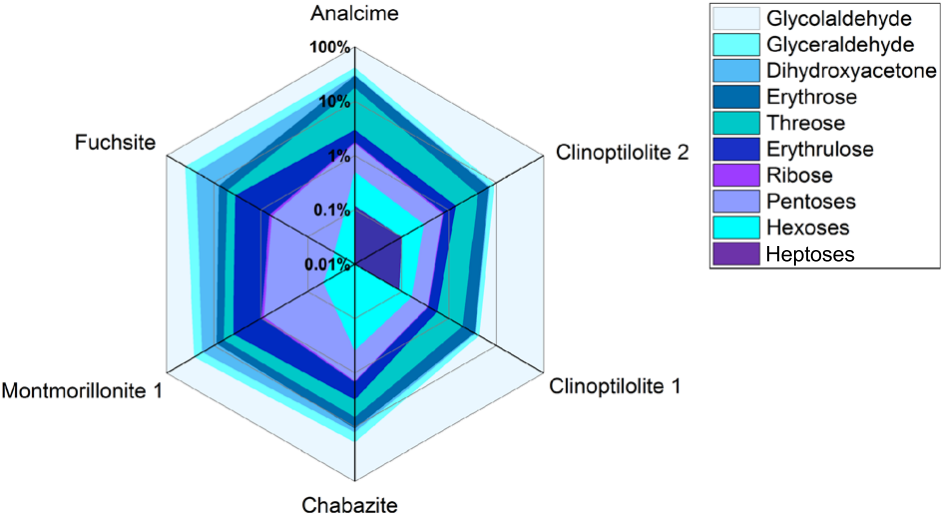
Incorporation of adsorbed formaldehyde in the mechanochemical formose reaction. Comparison of product distribution observed after ball milling of formaldehyde adsorbed onto minerals with 1:1 glycolaldehyde (head space gas: nitrogen, planetary ball mill, 90 min, 400 rpm). Analysis of products after derivatisation as silylated *O*-ethyl oximes by GC–MS. Depicted are mean values of product ratio percentages, individual data points are omitted for clarity (for detailed tabulated data see Supplementary Table 5).

In the next step, **1** was adsorbed on catalytically active minerals, that are also suitable as a support. Mineral adsorption of atmospheric form-aldehyde could have played a significant role on the early Earth^34^. From the available minerals, zeolites and sheet silicates were selected. All showed adsorption capacity and the incorporation of adsorbed **1** when reacting it with **2** in a planetary ball mill for 90 min at 400 rpm. Due to the use of **1**, the product set extended to glyceraldehyde **3a**, dihydroxyacetone **3b**, ribose and other pentoses **5** and even heptoses (Fig. 3). As the mineral supports are already catalysts themselves, no addition of further catalysts is needed. When calcium hydroxide was added the conversion increased, as did the amount of side products. Methanol **8**, glycolic acid **9**, lactic acid **12**, glycerol **15** and branched sugars, e.g. apiose **17** were detected. GC chromatograms and mass spectra of reference compounds and assigned reaction products can be found in the Supplementary Information (Supplementary Figs. 2–11). Formic acid **7**, one of the Cannizzaro products formed from **1**, was not detected. Whether it was not formed or directly reacted further could not be determined. Without the addition of **2** the umpolung of **1** was not accomplished and no conversion was observed.

In order to achieve the reaction starting from formaldehyde, other formose catalysts were tested for **1** adsorbed on molecular sieves. 2-Hydroxy-1-phenylethan-1-one as an enolisable compound is known to enhance the reaction in aqueous environments^15^. It reacts with **1** in an aldol reaction itself and subsequent retro-aldol reactions can produce sugars. Even though in the mechanochemical setting retro-aldol reactions only occurred in minute amounts, traces of sugars **3** and **4** were detected (Supplementary Fig. 1). In addition, thiazolium salts, that are known for their capability to catalyse the umpolung reaction were also probed for their activity in the mechanochemical reaction. 3-Ethylbenzothiazoliumbromide, 3-ethylthiazoliumbromide and 3-methylbenzothiazoliumiodide were chosen. Whereas with the two ethyl-substituted thiazolium salts traces of **3** and **4** were built from **1** again (Supplementary Fig. 1), the use of 3-methylbenzothiazoliumiodide produced no sugar formation. This catalyst also showed less activity in the aqueous reaction than the corresponding ethyl-substituted thiazolium salt^14^.

### Atmospheric compatibility

**Fig. 4:**
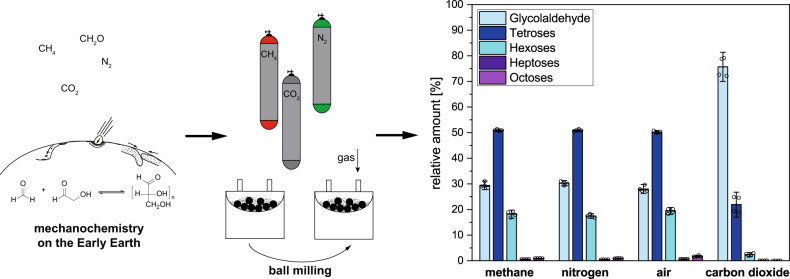
Influence of different atmospheres. Transfer of possible early Earth conditions and geochemical setting to laboratory conditions and comparison of product distribution observed after ball milling of glycolaldehyde and calcium hydroxide under methane, nitrogen, air or carbon dioxide gas phases (20 mol% catalyst, planetary ball mill, 90 min, 400 rpm). Analysis of products after derivatisation as silylated *O*-ethyl oximes by GC–MS. Depicted are mean values with standard deviations as well as individual data points of double reactions and double derivatisation and analyses (for detailed tabulated data see Supplementary Table 6).

There is much debate on the composition and the oxidation state of the early Earth’s atmosphere. As gases like oxygen inhibit the reaction in water^35^, we examined the effect of different atmospheres on the mechanochemical reaction. **2** and calcium hydroxide were milled for 90 min at 400 rpm in a planetary ball mill after evacuation and refilling the milling jars with the desired gas. The reaction under air, nitrogen and methane did not differ in the conversion and product distribution (Fig. 4). When carbon dioxide was used, the conversion dropped significantly from about 85–40%, but still the desired monosaccharides were formed, and no additional side products were observed.

## Discussion

We have demonstrated that the mechanochemical sugar formation starting from glycolaldehyde is catalysed by a variety of mineral classes including hydroxides, carbonates, silicates, micas, zeolites, clays, olivines, phosphates and phosphides. In a rather short reaction time, the solid-phase reaction uniformly generates monosaccharides, which could be enriched over time. In contrast to the aqueous reaction substantially reduced side and decomposition reactions are observed. The product distribution can be altered by the chosen mineral in favour of not only aldoses or ketoses, but also individual sugars. Even in the same mineral class the reactivity and selectivity of the reaction can be significantly modified by changes in the composition of cations.

Unlike under aqueous conditions, the reaction is also non-sensitive towards oxygen. It proceeds under different gases and under a reducing or oxidising atmosphere. Only for carbon dioxide the conversion decreased, which is likely due to carbonate formation and decrease of the alkalinity of the reaction mixture. Carbonate formation from carbon dioxide gas is not only known to occur in aqueous solution, but has also been observed previously for a mechanochemical setting^36^.

In addition, formaldehyde was adsorbed on zeolites and sheet silicates and as these are catalysts in the formose network themselves, the mechanochemical reaction with the addition of glycolaldehyde incorporates formaldehyde building valuable sugar products like glyceraldehyde, dihydroxyacetone and pentoses like ribose. Small amounts of side products were formed, such as lactic acid or apiose, which are also relevant in today’s metabolism and plant cell walls^37^.

The umpolung reaction necessary when starting from form-aldehyde did not occur mechanochemically with mineral catalysis. However prebiotic sources of glycolaldehyde have also been proposed^34,38^ and can initiate the reaction. Moreover, the addition of umpolung catalysts like thiazolium salts, which could correspond to precursors of today’s vitamin B_1_ thiamine, led to the formation of sugars. The missing activity of the methyl-substituted thiazolium salt is in agreement with its lesser activity compared to the other thiazolium salts in water and could be due to the substituents influence on the catalyst’s nucleophilicity^14^.

Overall, the mechanochemical formose reaction provides a robust synthesis route for monosaccharides in a geochemical setting likely to have existed on the early Earth. In addition, the solid-phase reaction offers an extra-terrestrial pathway to sugars independent of aqueous environments accounting for the recent finds of sugars in meteorites even without aqueous alteration phases.

## Methods

### Materials

All chemicals have been purchased from commercial suppliers in analytical grade and used without further purification. Minerals have been purchased from mineral dealers or have been supplied by the Department for Earth and Environmental Sciences of the LMU Munich. For a detailed list of minerals, their composition and particle size see Supplementary Tables 1, 3 and 7. Thiazolium salts have been synthesised according to literature procedures^14^. Para-formaldehyde was dried in a desiccator over phosphorus pentoxide, depolymerised at 150 °C under nitrogen flow and led through a column filled with dried mineral or molecular sieves (4 Å). The amount of adsorbed formaldehyde was determined by the weight difference of the support.

### Mechanochemical reactions

Catalytic activity of minerals was tested in the oscillatory ball mill CryoMill (Retsch GmbH, Haan, Germany) in 5 mL stainless steel milling jars with one 7 mm stainless steel ball. The total mass was kept constant (ca. 155 mg) to ensure equal energy input (for original sample weights see Supplementary Table 2). Glycolaldehyde-dimer (0.5 eq.) and the mineral (0.2 eq.) were added to the milling jar and mixed at 30 Hz for 90 min at the room temperature or with liquid nitrogen cooling intermitted by cooling periods (15 min precooling, six cycles of 15 min grinding at 30 Hz plus 5 min cooling). Reactions involving formaldehyde or gas atmospheres were conducted in the planetary ball mill Pulverisette 7 premium line (Fritsch GmbH, Idar-Oberstein, Germany) in 20 mL stainless steel grinding bowls with ten 10 mm stainless steel balls and equipped with gassing lids. For the different atmospheres, glycolaldehyde-dimer (850 mg, 0.5 eq.) and calcium hydroxide (210 mg, 0.2 eq.) were added and the jar closed. The jar was evacuated thrice and filled with the desired gas up to atmospheric pressure. For reactions with formaldehyde adsorbed on minerals, glycolaldehyde-dimer (0.5 eq.) and ads. formaldehyde (1.0 eq.) were added to the grinding bowls. For umpolung reactions, ads. formaldehyde (125 mg, 1.0 eq.), calcium hydroxide (61.7 mg, 0.2 eq.) and the catalyst (0.2 eq.) were mixed. After closing, the bowls were flushed with nitrogen to avoid explosive formaldehyde/air mixtures. Milling was conducted at 400 rpm for 90 min. All samples were stored at −78 °C prior to analysis.

### Derivatization and analysis

Samples were derivatised as silylated oximes in a two-step procedure using *O*-ethyl hydroxylamine and *N-/O-*bistrifluoroacetamide with the internal standard phenyl-β-D-glucopyranoside following a previously described method^39^. Separation and analysis was performed by gas chromatography–mass spectrometry (GC–MS) on a SE-52 column (L = 14 m, ID = 250 µm, FT = 250 nm) using a Thermo Trace GC Ultra coupled to a flame ionisation detector and a PolarisQ mass spectrometer in electron impact mode (EI). Identification was performed in comparison to the retention time and EI mass spectrum of reference compounds. Quantification was performed using the FID trace and after correcting the relative response using the effective carbon number^40^. If not stated otherwise, the depicted values are mean values with error values as standard mean deviations of four data points arising from double reactions and for these each double derivatisation steps. Due to the high volatility, detection of formaldehyde, methanol and formic acid was conducted in a different manner: 20 mg of sample was suspended in 100 µL dichloromethane and thoroughly vortexed. The solid was removed via centrifugation and 0.5 µL of the supernatant was injected into a Thermo Trace 1310 GC coupled to a thermal conductivity detector (TCD). The separation was performed using a CP-Sil 5CP column (L = 50 m, ID = 320 µm, FT = 5 µm). The inlet was kept at 250 °C operated in split mode with a flow of 20 mL/min. The oven was kept constant at 50 °C and helium was used as carrier gas with 2 mL/min. The TCD was kept at 200 °C. Identification was performed in reference to the retention time of commercially bought standards (Supplementary Figs. 2–11).

## Supplementary information

Supplementary Information

## Data Availability

The data that support the findings of this study are available in the Supplementary Information or from the corresponding author on reasonable request.
