# Supplementary material for: Mineral-mediated carbohydrate synthesis by mechanical forces in a primordial geochemical setting
Source: Commun Chem. 2020 Oct 16;3:140. doi: 10.1038/s42004-020-00387-w (PMC9814773; doi:10.1038/s42004-020-00387-w)
Supplement: Supplementary file 1 — Supplementary Information [file 42004_2020_387_MOESM1_ESM.pdf]

Supplementary Information

Mineral-mediated carbohydrate synthesis by mechanical forces in a primordial geochemical setting

Maren Haas[1,2], Saskia Lamour[1], Sarah Babette Christ[1], Oliver Trapp[1,2 *]

[1] Department of Chemistry and Pharmacy, Ludwig-Maximilians-University, Butenandtstr. 5-13, 81377 Munich, Germany

[2] Max-Planck-Institute for Astronomy, Königstuhl 17, 69117 Heidelberg, Germany

[*]Correspondence: oliver.trapp@cup.uni-muenchen.de

Contents:

Minerals.. 2

Tabulated product distributions.. 3

Extracted ion chromatograms.. 6

GC-TCD chromatograms.. 7

Mass spectra.. 8

Minerals

Supplementary Table 1: *Source and origin of tested minerals.*

**Mineral**	**Class**	**Source**	**Origin (if known)**
Fuchsite<br>$(K,Cr)Al_2[AlSi_3O_{10}(OH)_2]$	mica	Mineralienhandel Dipl. Geol. A. Spinn, Erlangen, Germany	Brazil
Calcite<br>$Ca[CO_3]$	carbonate	Mineralienhandel Dipl. Geol. A. Spinn, Erlangen, Germany	-
Muskovite<br>$KAl_2[AlSi_3O_{10}(OH)_2]$	mica	Mineralienhandel Dipl. Geol. A. Spinn, Erlangen, Germany	Brazil
Analcime<br>$Na_2[Al_2Si_4O_{12}]\cdot 2H_2O$	zeolite	Alfa Aesar, Haverhill, USA	-
Colemanite<br>$Ca[B_3O_4(OH)_3]\cdot H_2O$	borate	Seltene Mineralien, Gunnar Färber, Samswegen, Germany	USA, California, Borax Mine
Anhydrite<br>$Ca[SO_4]$	sulphate	Seltene Mineralien, Gunnar Färber, Samswegen, Germany	Germany, Kohnstedt Steinbruch
Apatite<br>$Ca_5[(OH)(PO_4)_3]$	phosphate	abcr, Karlsruhe, Germany	-
Quartz<br>SiO_2	silicate	Seltene Mineralien, Gunnar Färber, Samswegen, Germany	-
Magnesite<br>$Mg[CO_3]$	carbonate	Mineralienhandel Dipl. Geol. A. Spinn, Erlangen, Germany	-
Portlandite<br>$Ca(OH)_2$	hydroxide	Seltene Mineralien, Gunnar Färber, Samswegen, Germany	Germany, Caspar Steinbruch
Brucite<br>$Mg(OH)_2$	hydroxide	Seltene Mineralien, Gunnar Färber, Samswegen, Germany	Pakistan, Kharan
Diaspore<br>$AlO(OH)$	hydroxide	Seltene Mineralien, Gunnar Färber, Samswegen, Germany	Turkey, Selcuk
Peridot<br>$(Mg,Fe)_2SiO_4$	olivine	Seltene Mineralien, Gunnar Färber, Samswegen, Germany	Pakistan, Sapat Gali
Montmorillonite 1<br>$(Na,Ca)_{0,3}[(Al,Mg)_2Si_4O_{10}(OH)_2]\cdot nH_2O$	clay	Alfa Aesar, Haverhill, USA	-
Montmorillonite 2<br>$(Na,Ca)_{0,3}[(Al,Mg)_2Si_4O_{10}(OH)_2]\cdot nH_2O$	clay	abcr, Karlsruhe, Germany	-
Talc<br>$Mg_3[(OH)_2Si_4O_{10}]$	silicate	Lithos, Ennsdorf, Austria	Afghanistan, Khugiani
Basalt<br>(pyroxene, plagioclase, olivine)	volcanic rock	Bettina Scheu, LMU Munich	Iceland, Krafla
Clinoptilolite 1<br>$(K,Na,Ca)_6[Si_{30}Al_6O_{72}]\cdot nH_2O$	zeolite	Zeocem, Bystré, Slovakia	Slovakia, Nižný Hrabovec
Clinoptilolite 2<br>$(K,Na,Ca)_6[Si_{30}Al_6O_{72}]\cdot nH_2O$	zeolite	Lithos, Ennsdorf, Austria	Romania, Zalau
Chabazite<br>$(Ca,K,Mg,Na)_2[Al_4Si_8O_{24}]\cdot nH_2O$	zeolite	St. Cloud Mining, Tucson, USA	USA, Arizona, Bowie
Schreibersite<br>$(Fe,Ni,Co)_3P$	phosphide	Sigma Aldrich, St. Louis, USA	synthetic

Supplementary Table 2: Weighted sample masses for the mineral-catalysed aldol reaction starting from glycolaldehyde-dimer (GA-D) and 20 mol% catalyst.

**Mineral**	**M_{Min} [g/mol]**	**m_{Min} [mg]**	**$m_{GA\text{-}D}$ [mg]**
Fuchsite<br>$(K,Cr)Al_2[AlSi_3O_{10}(OH)_2]$	404.8	84.3	66.0
Calcite<br>$Ca[CO_3]$	100.1	38.3	115.0
Muskovite<br>$KAl_2[AlSi_3O_{10}(OH)_2]$	397.8	86.2	65.0
Analcime<br>$Na_2[Al_2Si_4O_{12}]\cdot 2H_2O$	440.3	89.0	60.0
Colemanite<br>$Ca[B_3O_4(OH)_3]\cdot H_2O$	205.5	61.6	90.0
Anhydrite<br>$Ca[SO_4]$	136.1	47.6	105.0
Apatite<br>$Ca_5[(OH)(PO_4)_3]$	502.3	92.0	55.0
Quartz<br>SiO_2	60.1	25.0	125.0
Magnesite<br>$Mg[CO_3]$	84.3	33.7	120.0
Portlandite<br>$Ca(OH)_2$	74.1	30.8	125.0
Brucite<br>$Mg(OH)_2$	58.3	24.3	125.0
Diaspore<br>$AlO(OH)$	60.0	25.0	125.0
Peridot<br>Mg_2SiO_4	140.7	49.2	105.0
Montmorillonite 1<br>$Na_{0,3}[(Al,Mg)_2Si_4O_{10}(OH)_2]\cdot H_2O$	385.9	90.0	70.0
Montmorillonite 2<br>$Na_{0,3}[(Al,Mg)_2Si_4O_{10}(OH)_2]\cdot H_2O$	385.9	90.0	70.0
Talc<br>$Mg_3[Si_4O_{10}(OH)_2]$	379.3	88.4	70.0
Basalt<br>(pyroxene, plagioclase, olivine)	*	75.0	75.0
Clinoptilolite 1<br>$Na_6[AlSi_5O_{12}]\cdot H_2O$		75.0	75.0
Clinoptilolite 2<br>$(K,Na,Ca)_6[Si_{30}Al_6O_{72}]\cdot nH_2O$		75.0	75.0
Chabazite<br>$Ca_2[Al_4Si_8O_{24}]\cdot 13H_2O$		75.0	75.0
Schreibersite<br>Fe_3P	198.5	59.5	90.0

*as the volcanic rock persists of several components, no molar mass was estimated

Supplementary Table 3: Elemental analysis results of the mineral catalysts.

**Mineral**	**Method**	**Result**
Fuchsite	ICP-OES	Al: 122.47 mg/g, Ca: 0.18 mg/g, Cr: 7.67 mg/g, Fe: 2.98 mg/g, K: 41.42 mg/g, Mg: 1.44 mg/g, Mn: 0.02 mg/g, Na: 0.79 mg/g, Si: 181.05 mg/g
Calcite	ICP-OES	B: 0.11 mg/g, Ca: 395.65 mg/g, Fe: 0.01 mg/g, K: 0.32 mg/g, Mg: 2.50 mg/g, Mn: 0.15 mg/g, Na: 0.08 mg/g, S: 0.84 mg/g, Si: 0.99 mg/g
Muscovite	ICP-OES	Al: 74.73 mg/g, Fe: 1.36 mg/g, K: 27.60 mg/g, Mg: 0.07 mg/g, Mn: 0.07 mg/g, Na: 4.82 mg/g, Si:92.47 mg/g
Analcime	ICP-OES	Al: 115.80 mg/g, Ca: 0.60 mg/g, K: 4.10 mg/g, Mg: 0.59 mg/g, Mn: 4.84 mg/g, Na: 72.96 mg/g, Si: 244.11 mg/g
Colemanite	ICP-OES	Al: 5.28 mg/g, B: 141.59 mg/g, Ca: 198.12 mg/g, Fe: 1.90 mg/g, K: 2.44 mg/g, Mg: 2.49 mg/g, Mn: 0.71 mg/g, Na: 1.18 mg/g, S: 4.15 mg/g, Si: 16.31 mg/g
Anhydrite	ICP-OES	B: 0.34 mg/g, Ca: 292.25 mg/g, K: 0.57 mg/g, Mg: 2.08 mg/g, Na: 0.17 mg/g, S: 233.89 mg/g, Si: 0.91 mg/g
Apatite	ICP-OES	Ca: 380.41 mg/g, Mg: 0.30 mg/g, Mn: 0.10 mg/g, Na: 0.98 mg/g, P:185.85 mg/g
Quartz	ICP-OES	Si: 273.46 mg/g
Magnesite	ICP-OES	B: 0.07 mg/g, Ca: 4.72 mg/g, Fe: 0.07 mg/g, K: 0.35 mg/g, Mg: 289.00 mg/g, Mn: 0.07 mg/g, Na: 0.08 mg/g, S: 0.35 mg/g, Si: 4.60 mg/g
Portlandite	ICP-OES	B: 0.07 mg/g, Ca: 514.18 mg/g, Fe: 1.73 mg/g
Brucite	ICP-OES	B: 0.15 mg/g, Fe: 2.20 mg/g, Mg 433.99 mg/g
Diaspore	SEM-EDX	Al: 34%, O: 66%
Peridot	ICP-OES	Cr: 0.08 mg/g, Fe: 12.25 mg/g, Mg: 114.36 mg/g, Mn: 1.05 mg/g, Si: 178.48 mg/g
Montmorillonite 1	ICP-OES	Al: 88.71 mg/g, Ca: 7.63 mg/g, Fe: 24.38 mg/g, K: 1.09 mg/g, Mg: 13.84 mg/g, Mn: 0.22 mg/g, Na: 9.83 mg/g, Si: 260.00 mg/g
Montmorillonite 2	ICP-OES	Al: 82.73 mg/g, Ca: 11.44 mg/g, Fe: 22.02 mg/g, K 2.01 mg/g, Mg: 29.93 mg/g, Na: 21.00 mg/g, Si: 244.01 mg/g, Ti: 1.37 mg/g
Talc	ICP-OES	Al: 3.41 mg/g, Ca: 5.02 mg/g, Fe: 3.97 mg/g, Mg 200.00 mg/g, Si: 281.31 mg/g
Basalt	ICP-OES	Al: 67.53 mg/g, Ca: 71.99 mg/g, Fe: 114.91 mg/g, K: 2.57 mg/g, Mg: 35.62 mg/g, Mn: 1.93 mg/g, Na: 12.79 mg/g, Si: 228.79 mg/g, Sr: 0.16 mg/g, Ti: 12.13 mg/g, B: 0.09 mg/g, Cr: 0.13 mg/g, P: 0.88 mg/g, S: 1.63 mg/g
Clinoptilolite 1	ICP-OES	Al: 62.80 mg/g, Ca: 20.80 mg/g, Fe: 8.51 mg/g, K: 28.06 mg/g, Mg: 4.29 mg/g, Na: 3.51 mg/g, Si: 212.10 mg/g, Sr: 0.21 mg/g, Ti: 0.80 mg/g
Clinoptilolite 2	ICP-OES	Al: 60.97 mg/g, Ca: 22.60 mg/g, Fe: 10.00 mg/g, 24.56 mg/g, Mg: 6.01 mg/g, Mn: 0.24 mg/g, Na: 9.87 mg/g, Si: 302.39 mg/g, Sr: 0.41 mg/g, Ti: 0.73 mg/g
Chabazite	ICP-OES	Al: 69.90 mg/g, Ca: 13.53 mg/g, Fe: 21.13 mg/g, K: 9.17 mg/g, Mg: 11.74 mg/g, Mn: 0.16 mg/g, Na: 26.06 mg/g, Si: 253.93 mg/g, Sr: 1.30 mg/g, Ti: 1.08 mg/g
Schreibersite	SEM-EDX	synthetic material, characterisation see New J. Phys. 2018, 20, 055003.

Tabulated data

Supplementary Table 4: *Product distribution of mineral-catalysed mechanochemical monosaccharide formation starting from glycolaldehyde.*

Mineral	Apatite		Magnesite		Calcite		Anhydrite		Diaspore		Brucite		Portlandite	
**Products [%]**		±		±		±		±		±		±		±
**Glycolaldehyde**	92.38	0.31	99.03	0.09	96.86	1.19	100.00	0.00	99.89	0.01	92.73	0.99	44.93	1.33
**Erythrose**	3.19	0.14	0.39	0.04	1.27	0.50	0.00	0.00	0.05	0.01	2.71	0.37	15.79	0.42
**Threose**	3.69	0.13	0.42	0.05	1.44	0.59	0.00	0.00	0.06	0.01	3.80	0.52	21.71	0.44
**Erythrulose**	0.30	0.02	0.06	0.01	0.12	0.02	0.00	0.00	0.00	0.00	0.28	0.04	5.42	0.15
**Hexoses**	0.44	0.07	0.10	0.03	0.31	0.11	0.00	0.00	0.00	0.00	0.47	0.07	12.16	0.35
**Conversion**	14.51		2.02		6.36		0.00		0.22		13.93		73.13	

Mineral	Montmoril. 2		Montmoril. 1		Clinoptilolite 2		Clinoptilolite 1		Analcime		Chabazite		Schreibersite	
**Products [%]**		±		±		±		±		±		±		±
**Glycolaldehyde**	85.26	2.86	96.55	0.92	98.51	0.08	98.93	0.08	97.08	0.70	97.21	0.77	99.15	0.08
**Erythrose**	6.17	0.98	1.68	0.44	0.65	0.05	0.44	0.01	1.11	0.27	1.40	0.41	0.36	0.03
**Threose**	7.28	1.51	1.37	0.35	0.65	0.03	0.47	0.02	1.49	0.37	1.25	0.37	0.37	0.03
**Erythrulose**	0.74	0.19	0.24	0.10	0.13	0.03	0.12	0.02	0.17	0.04	0.05	0.01	0.03	0.02
**Hexoses**	0.55	0.19	0.16	0.06	0.05	0.02	0.04	0.04	0.15	0.05	0.09	0.03	0.09	0.03
**Conversion**	26.04		6.82		2.96		2.17		5.82		5.51		1.77	

Mineral	Basalt		Colemanite		Peridot		Talc		Quartz		Muskovite		Fuchsite	
**Products [%]**		±		±		±		±		±		±		±
**Glycolaldehyde**	99.03	0.17	100.00	0.00	99.05	0.17	84.55	4.40	99.89	0.02	98.38	0.23	96.69	1.73
**Erythrose**	0.33	0.05	0.00	0.00	0.38	0.05	7.04	2.01	0.05	0.01	0.70	0.09	1.40	0.76
**Threose**	0.41	0.06	0.00	0.00	0.44	0.08	6.93	1.91	0.05	0.01	0.72	0.10	1.38	0.68
**Erythrulose**	0.14	0.02	0.00	0.00	0.05	0.03	0.80	0.21	0.00	0.00	0.20	0.03	0.51	0.27
**Hexoses**	0.09	0.05	0.00	0.00	0.08	0.04	0.68	0.26	0.00	0.00	0.00	0.00	0.01	0.01
**Conversion**	2.00		0.00		1.95		27.19		0.21		3.20		6.41	

Supplementary Table 5: *Product distribution of mineral-catalysed mechanochemical monosaccharide formation starting from glycolaldehyde and adsorbed formaldehyde.*

**Mineral**	**Fuchsite**		**Montmorillonite 1**		**Chabazite**		**Clinoptilolite 1**		**Clinoptilolite 2**		**Analcime**	
**Amount of ads. 1**	1.1 wt.%		3.1 wt.%		10.8 wt.%		9.7 wt.%		8.6 wt.%		2.5 wt.%	
**Products [%]**		±		±		±		±		±		±
**Glycolaldehyde**	60.22	0.29	74.28	0.21	81.11	0.23	95.78	0.02	91.19	0.07	59.15	0.21
**Glyceraldehyde**	16.34	0.09	8.46	0.01	6.59	0.06	0.69	0.02	1.76	0.01	11.39	0.03
**Dihydroxyacetone**	15.78	0.08	8.82	0.15	1.82	0.03	0.15	0.01	0.40	0.01	0.83	0.00
**Erythrose**	2.63	0.02	2.41	0.02	4.03	0.06	1.47	0.01	2.77	0.02	11.38	0.07
**Threose**	1.63	0.01	2.27	0.03	3.40	0.05	1.36	0.02	2.54	0.03	14.39	0.08
**Erythrulose**	2.76	0.01	2.81	0.04	1.60	0.01	0.19	0.01	0.64	0.01	1.17	0.01
**other Pentoses**	0.54	0.06	0.80	0.03	0.99	0.02	0.19	0.01	0.42	0.01	1.16	0.02
**Ribose**	0.07	0.01	0.10	0.00	0.07	0.01	0.01	0.01	0.05	0.01	0.10	0.01
**Hexoses**	0.03	0.01	0.05	0.03	0.38	0.01	0.07	0.01	0.19	0.01	0.37	0.02
**Heptoses**	0.00	0.00	0.00	0.00	0.00	0.00	0.09	0.01	0.10	0.02	0.12	0.01

Supplementary Table 6: *Product distribution of mechanochemical monosaccharide formation starting from glycolaldehyde and calcium hydroxide under different atmospheres.*

**Atmosphere**	**Nitrogen**		**Carbon Dioxide**		**Air**		**Methane**	
**Products [%]**		±		±		±		±
**Glycolaldehyde**	30.24	0.59	75.66	3.28	28.00	0.99	29.41	0.97
**Erythrose**	18.08	0.20	9.21	1.14	17.67	0.15	17.76	0.14
**Threose**	25.63	0.14	11.77	1.53	25.27	0.12	25.42	0.17
**Erythrulose**	7.20	0.43	0.90	0.15	7.30	0.17	7.76	0.10
**Hexoses**	17.47	0.54	2.40	0.42	19.39	0.80	18.18	0.93
**Heptoses**	0.45	0.05	0.04	0.04	0.67	0.04	0.57	0.03
**Octoses**	0.93	0.07	0.02	0.02	1.69	0.24	0.91	0.04

Supplementary Table 7*: Particle size of each mineral before and after the ball mill reaction determined by optical microscopy.*

**Mineral**	**Particle Size [µm]**
Fuchsite	15-50
Calcite	50-110
Muscovite	15-50
Analcime	50-150
Colemanite	30-110
Anhydrite	80-190
Apatite	15-110
Quartz	15-80
Magnesite	15-80
Portlandite	30-110
Brucite	50-175
Diaspore	80-150
Peridot	30-110
Montmorillonite 1	15-80
Montmorillonite 2	15-50
Talc	8-15
Basalt	350-500
Clinoptilolite 1	80-250
Clinoptilolite 2	15-50
Chabazite	15-50
Schreibersite	50-400
All after milling	5-30

Extracted ion chromatograms

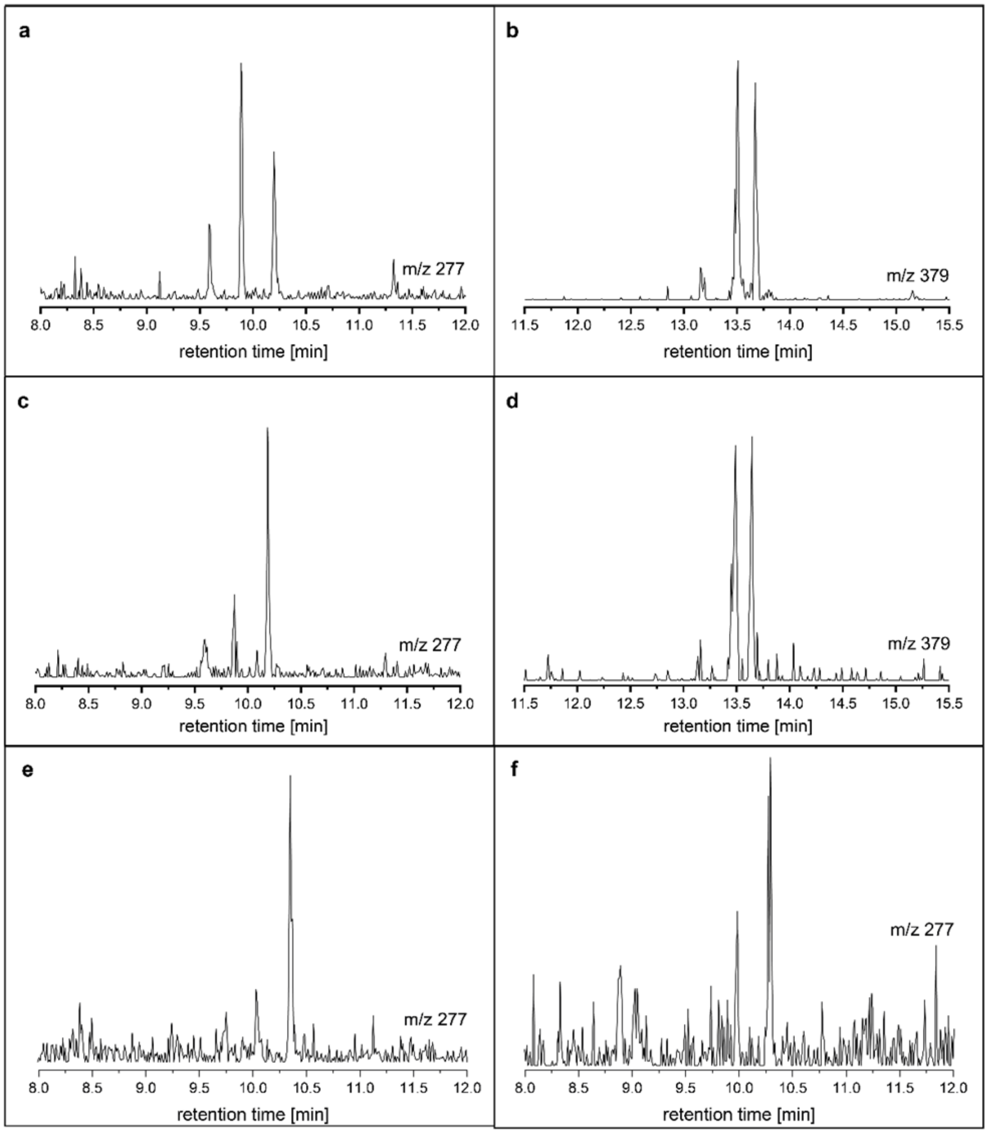

Supplementary Figure 1: *Extracted ion chromatograms of derivatized sugar samples of the mechanochemical reaction starting from formaldehyde adsorbed on molecular sieves, calcium hydroxide and* ***a/b*** *2-hydroxy-1-phenylethan-1-on,* ***c/d*** *3-ethylthiazoliumbromide,* ***e*** *3-ethylbenzothiazoliumbromide or* ***f*** *3-methylbenzothiazoliumiodide.* ***a/c/e/f*** *m/z 277 corresponds to trioses,* ***b/d*** *m/z 379 corresponds to tetroses.*

GC-TCD Chromatograms

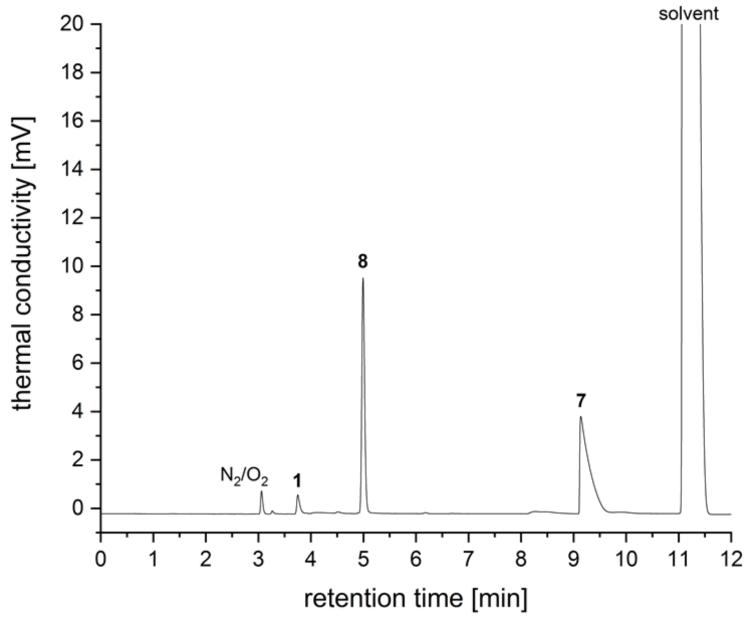

Supplementary Figure 2: *Gas chromatogram of reference compounds using a CP-Sil 5CP column (L = 50 m, ID = 320 µm, FT = 5 µm) at 50°C and 2 mL/min helium.* ***1*** *formaldehyde,* ***7*** *formic acid,* ***8*** *methanol.*

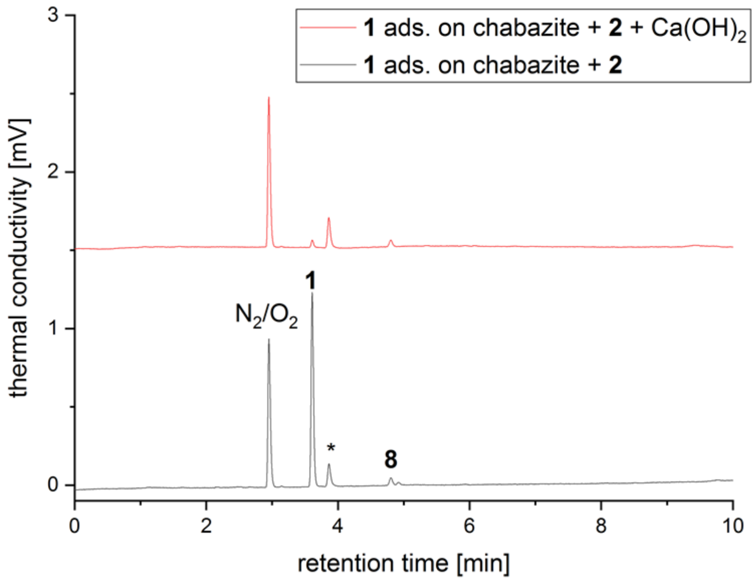

Supplementary Figure 3: *Gas chromatogram of mechanochemical reaction of* ***1*** *adsorbed on chabazite and* ***2*** *with and without additional calcium hydroxide catalyst forming* ***8*** *(*unidentified impurity from solvent).*

Mass spectra

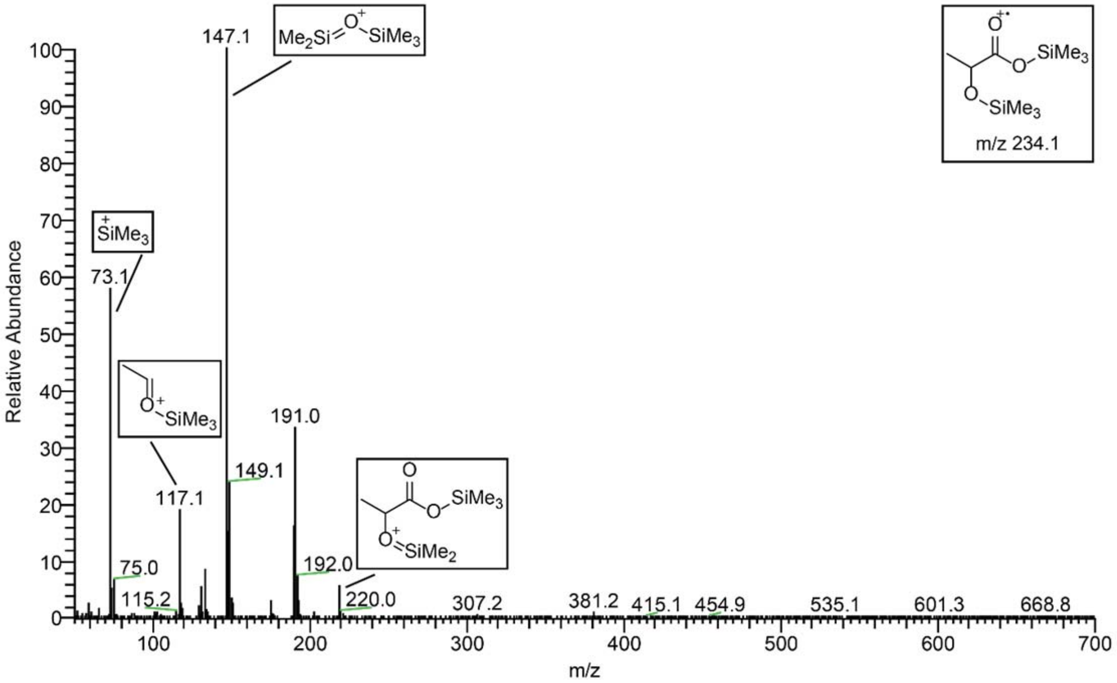

__Supplementary Figure 4:__ Reference mass spectrum for derivatized lactic acid __12__.

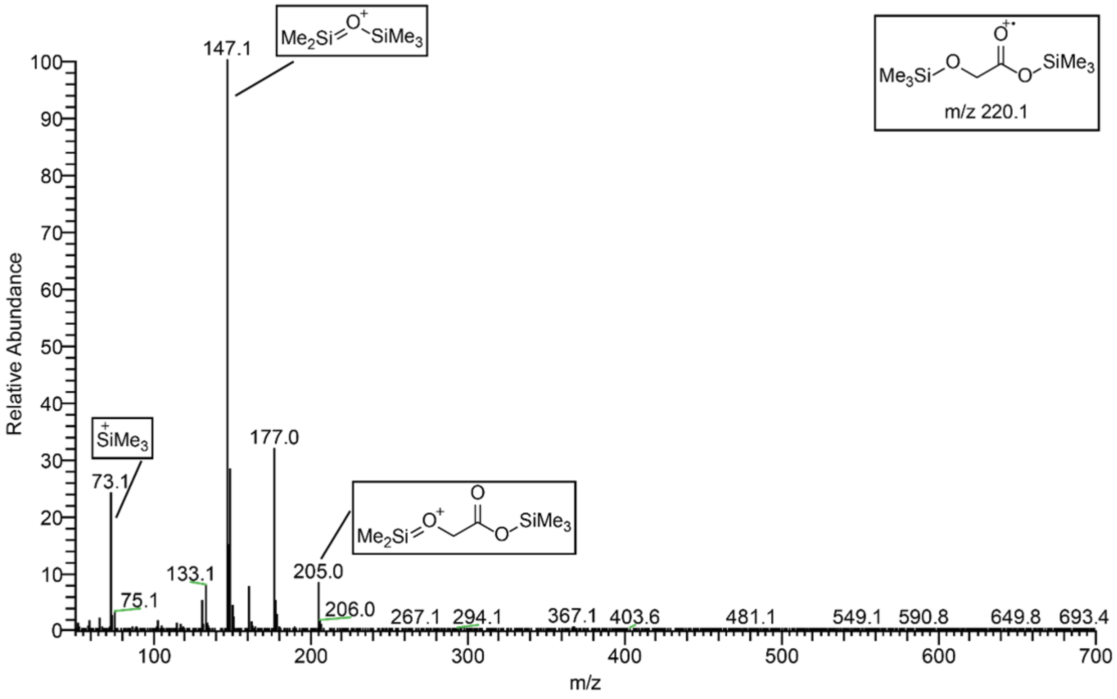

__Supplementary Figure 5:__ Reference mass spectrum for derivatized glycolic acid __9__.

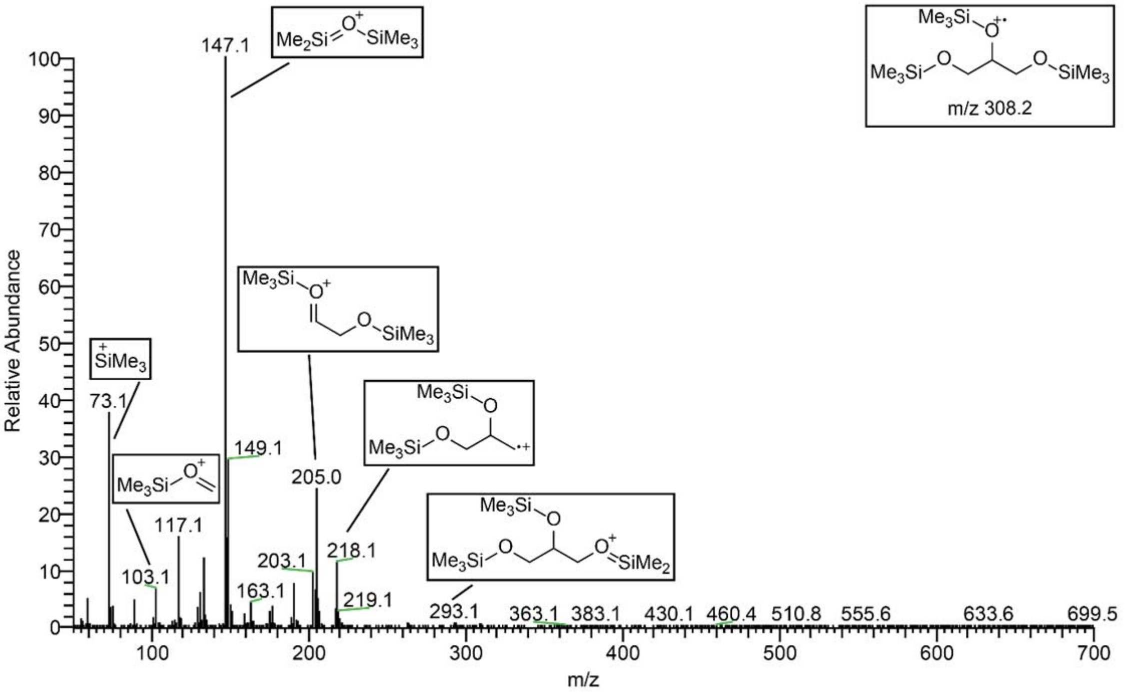

Supplementary Figure 6: *Reference mass spectrum of derivatized glycerol* ***15***.

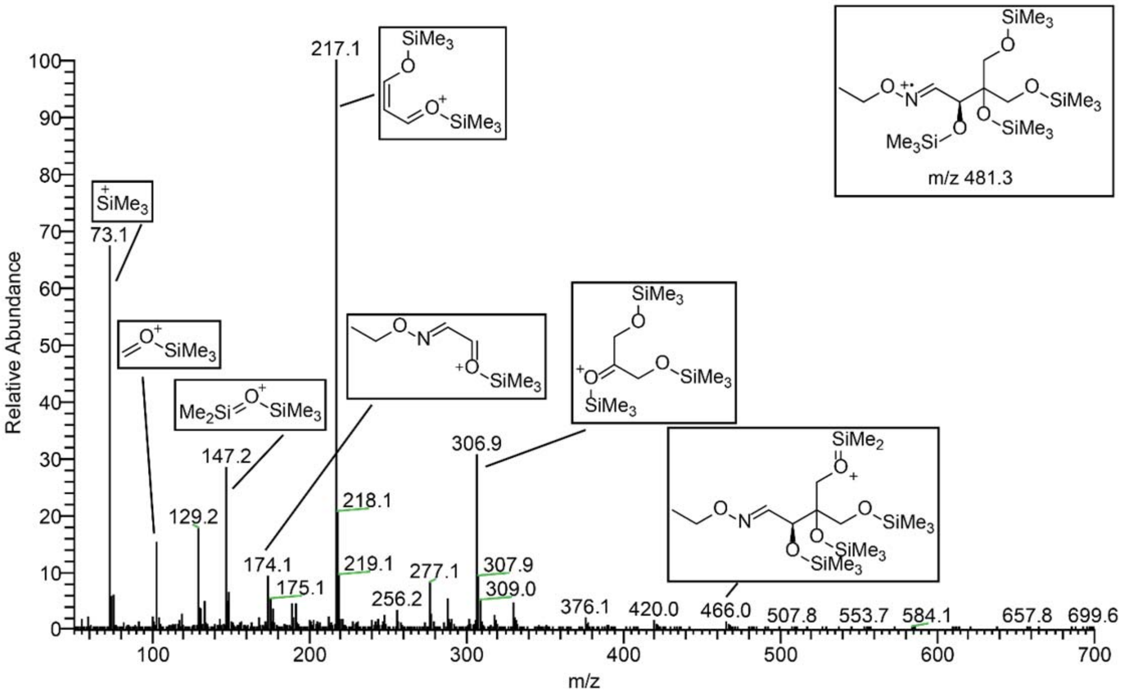

Supplementary Figure 7: *Reference mass spectrum of derivatized D-apiose* ***17***.

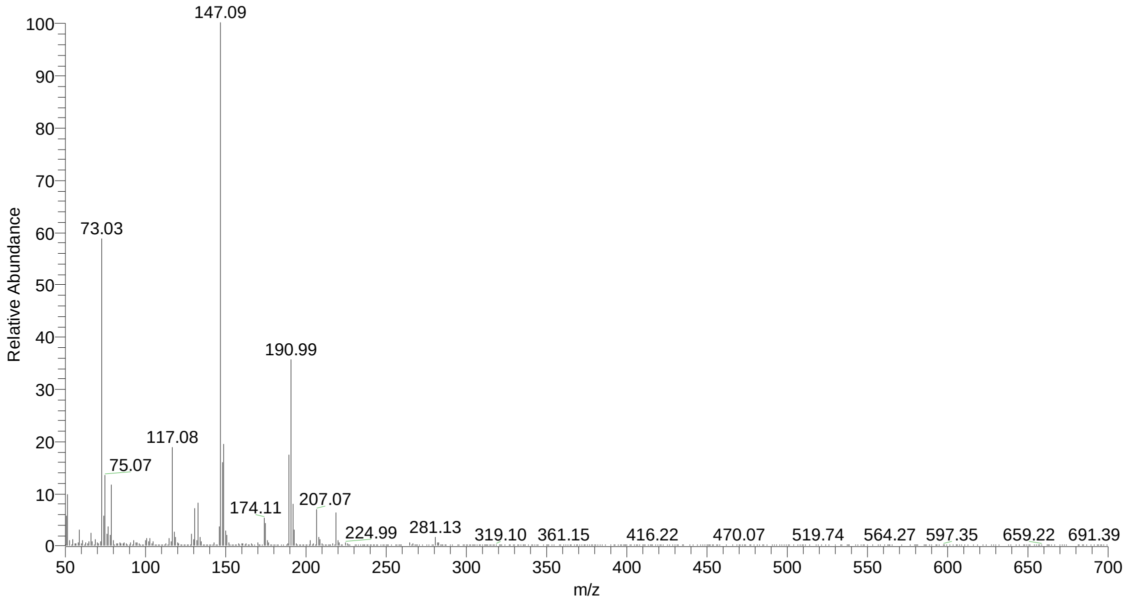

Supplementary Figure 8: *Mass spectrum of peak at* t_{ret} *= 6.4 min of mechanochemical reaction of adsorbed* ***1*** *on analcime +* ***2*** *+ calcium hydroxide after derivatization as trimethylsilylated* O*-ethyloximes. This peak corresponds to lactic acid* ***12****.*

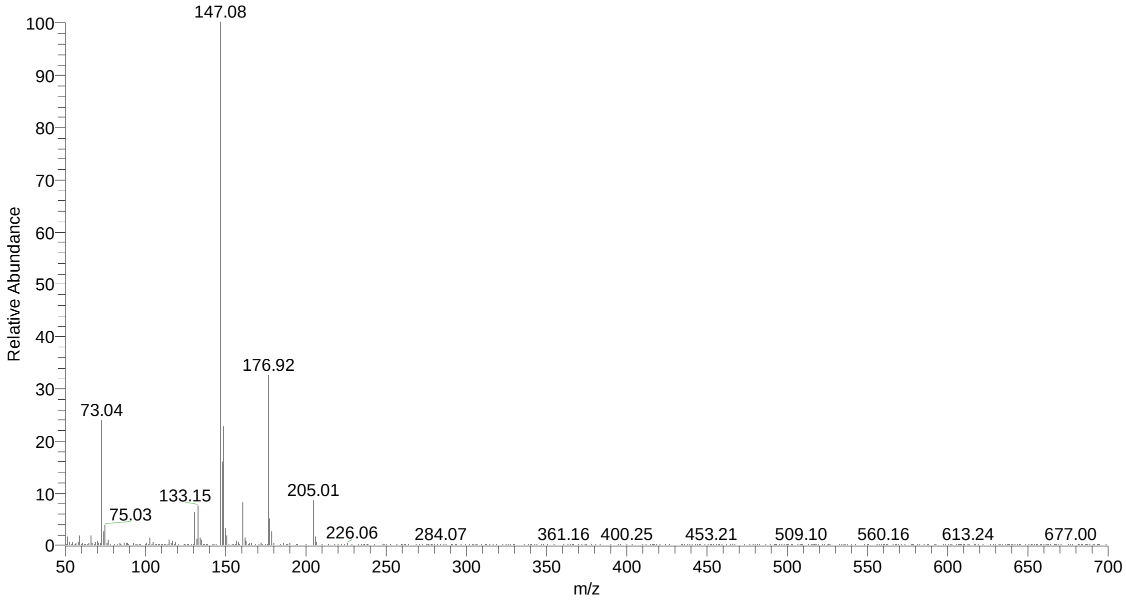

Supplementary Figure 9: *Mass spectrum of peak at* t_{ret} *= 6.6 min of mechanochemical reaction of adsorbed* ***1*** *on analcime +* ***2*** *+ calcium hydroxide after derivatization as trimethylsilylated* O*-ethyloximes. This peak corresponds to glycolic acid* ***9****.*

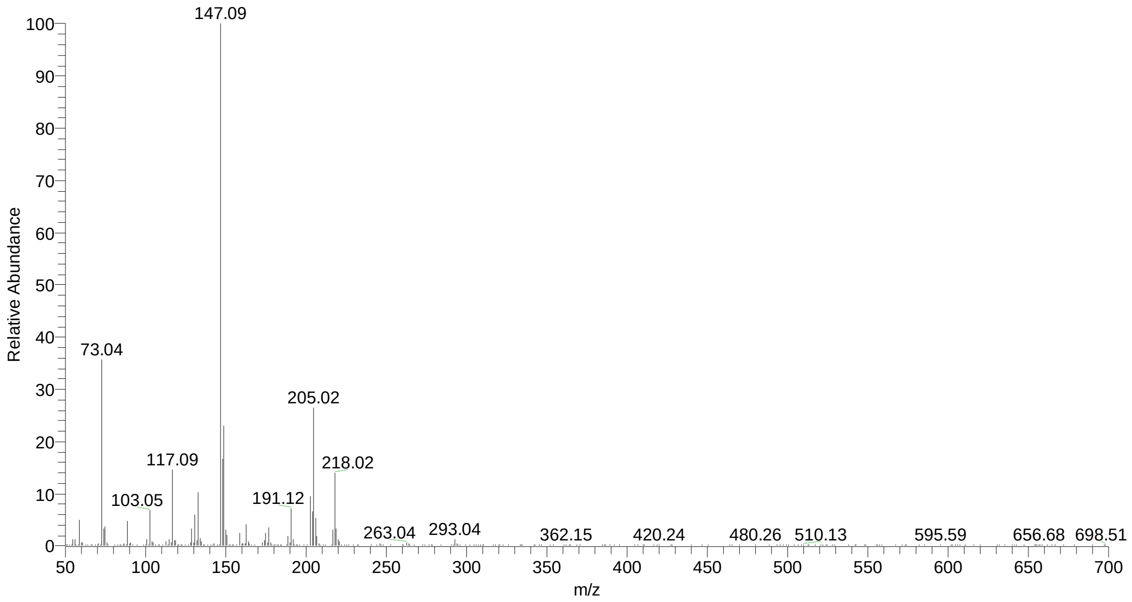

Supplementary Figure 10: *Mass spectrum of peak at t_{ret} = 9.8 min of mechanochemical reaction of adsorbed* ***1*** *on analcime +* ***2*** *+ calcium hydroxide after derivatization as trimethylsilylated* O*-ethyloximes. This peak corresponds to glycerol* ***15****.*

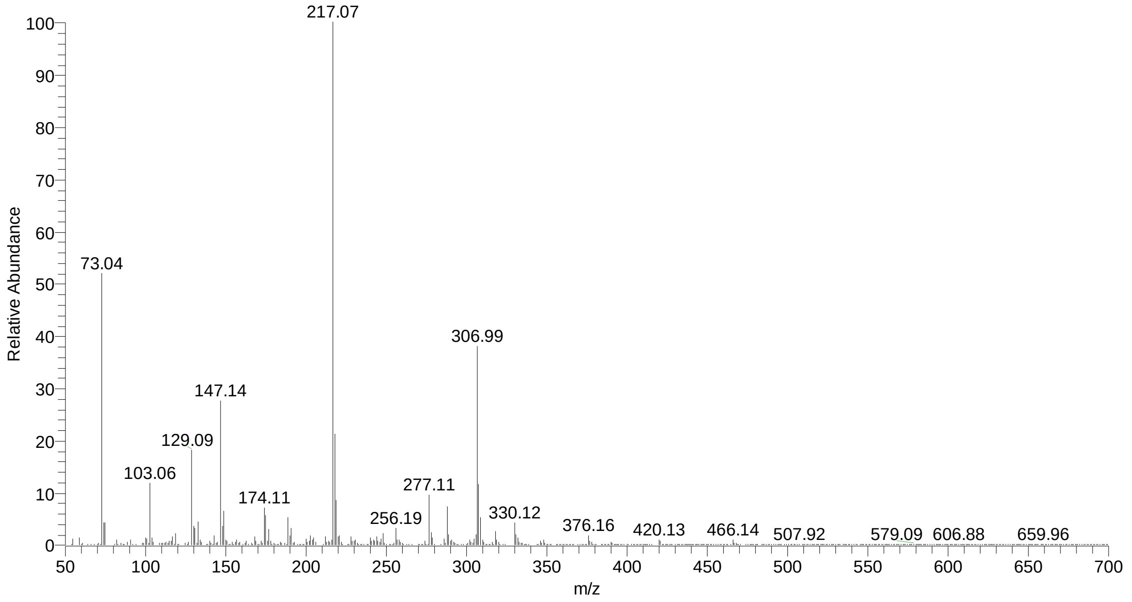

Supplementary Figure 11: *Mass spectrum of peak at t_{ret} = 16.8 min of mechanochemical reaction of adsorbed* ***1*** *on analcime +* ***2*** *+ calcium hydroxide after derivatization as trimethylsilylated* O*-ethyloximes. This peak corresponds to apiose* ***17****.*
